# The *Mycobacterium tuberculosis* transcriptional landscape under genotoxic stress

**DOI:** 10.1186/s12864-016-3132-1

**Published:** 2016-10-10

**Authors:** Amine Namouchi, Marta Gómez-Muñoz, Stephan A. Frye, Line Victoria Moen, Torbjørn Rognes, Tone Tønjum, Seetha V. Balasingham

**Affiliations:** 1 Department of Microbiology, Oslo University Hospital, Postboks 4950, NO-0424 Oslo, Norway; 2 Department of Microbiology, University of Oslo, Oslo, Norway; 3 Department of Informatics, University of Oslo, Oslo, Norway; 4 Current address: Department of Nutrition, University of Oslo, Oslo, Norway

**Keywords:** *Mycobacterium tuberculosis*, Transcriptomics, Genotoxic stress, RNA deep-sequencing, Non-coding RNA (ncRNA)

## Abstract

**Background:**

As an intracellular human pathogen, *Mycobacterium tuberculosis* (Mtb) is facing multiple stressful stimuli inside the macrophage and the granuloma. Understanding Mtb responses to stress is essential to identify new virulence factors and pathways that play a role in the survival of the tubercle bacillus. The main goal of this study was to map the regulatory networks of differentially expressed (DE) transcripts in Mtb upon various forms of genotoxic stress. We exposed Mtb cells to oxidative (H_2_O_2_ or paraquat), nitrosative (DETA/NO), or alkylation (MNNG) stress or mitomycin C, inducing double-strand breaks in the DNA. Total RNA was isolated from treated and untreated cells and subjected to high-throughput deep sequencing. The data generated was analysed to identify DE genes encoding mRNAs, non-coding RNAs (ncRNAs), and the genes potentially targeted by ncRNAs.

**Results:**

The most significant transcriptomic alteration with more than 700 DE genes was seen under nitrosative stress. In addition to genes that belong to the replication, recombination and repair (3R) group, mainly found under mitomycin C stress, we identified DE genes important for bacterial virulence and survival, such as genes of the type VII secretion system (T7SS) and the proline-glutamic acid/proline-proline-glutamic acid (PE/PPE) family. By predicting the structures of hypothetical proteins (HPs) encoded by DE genes, we found that some of these HPs might be involved in mycobacterial genome maintenance. We also applied a state-of-the-art method to predict potential target genes of the identified ncRNAs and found that some of these could regulate several genes that might be directly involved in the response to genotoxic stress.

**Conclusions:**

Our study reflects the complexity of the response of Mtb in handling genotoxic stress. In addition to genes involved in genome maintenance, other potential key players, such as the members of the T7SS and PE/PPE gene family, were identified. This plethora of responses is detected not only at the level of DE genes encoding mRNAs but also at the level of ncRNAs and their potential targets.

**Electronic supplementary material:**

The online version of this article (doi:10.1186/s12864-016-3132-1) contains supplementary material, which is available to authorized users.

## Background

Tuberculosis (TB) remains the leading cause of mortality due to a bacterial pathogen worldwide. The causative agent of TB, *Mycobacterium tuberculosis* (Mtb), infects approximately one third of the world’s human population [1]. The main route of Mtb infection is from the respiratory tract to the lung. After entering the lung, the first immune cell type encountered by the bacteria is the alveolar macrophage. Mtb has an extraordinary ability to persist and replicate inside the macrophages, which represent a hostile environment where most other pathogens perish. This ability is a prerequisite for Mtb to maintain infection. During latency, Mtb is viable but non-replicating and the bacteria are confined to granulomas [2]. The granuloma is a unique ecosystem where Mtb growth remains most of the time in balance with host antibacterial effector mechanisms [3].

After phagocytosis, Mtb experiences endogenous and exogenous oxidative stress [4]. The endogenous stress is the result of the incomplete reduction of molecular oxygen during aerobic respiration while the exogenous stress is due to the oxidative burst generated by NADPH oxidase in the phagosome [5]. The reactive oxygen species (ROS) that are created in these processes include peroxides, hydroxyl radicals and superoxide. In the macrophage, Mtb also faces nitrosative stress from reactive nitrogen species (RNS), such as nitric oxide, nitrite, nitrogen dioxide and nitrates [6]. ROS and RNS can be bactericidal as they react with a wide range of macromolecules, like nucleic acids, proteins, lipids and carbohydrates [7–9]. The tubercle bacillus counteracts the effects of ROS and RNS by producing several enzymes, including catalase, peroxidase, superoxide dismutase, and nitrosothiol reductase, to ensure its intracellular survival and persistence [10].

DNA double strand breaks (DSBs) are critical lesions that can result in cell death or a wide variety of genetic alterations. In mycobacteria, DSBs are repaired by three distinct repair mechanisms. These are homologous recombination (HR), dependent on AdnAB nucleases and RecA, non-homologous end-joining (NHEJ), involving KU and LigD [11], and single strand annealing (SSA), which is achieved through RecBCD in a RecA-independent manner [11].

Regulatory non-coding RNAs (ncRNAs) play important roles in bacterial gene regulation at the post-transcriptional level [12]. They are functional RNA molecules that are not translated into proteins. They usually have a length of 50–400 nucleotides and base-pair with mRNA to regulate mRNA stability or the efficiency of mRNA translation [13]. NcRNAs can be 5′ or 3′ untranslated regions (UTRs), antisense or *cis*-encoded small RNAs, and intergenic or *trans*-encoded transcripts [14]. Most ncRNAs regulate gene expression and can provide a quick response to changing environmental conditions such as genotoxic stress, nutrient deprivation or infection [15–17]. Studies on infectious processes have demonstrated that ncRNAs can coordinate precise gene expression and thus play an important role in microbial pathogenesis [16]. While the number of characterised ncRNAs steadily increases, only a limited number of the corresponding mRNA targets have been identified.

Until now, only a few studies reported on the global mycobacterial responses to genotoxic stress [6, 18] and, to our knowledge, there were no reports addressing the complexity of the mycobacterial response to several forms of genotoxic stress. Our main goal in this study was to explore the Mtb responses to various forms of genotoxic stress conditions by analysing the whole transcriptome. Mtb responses to oxidative agents (H_2_O_2_ and paraquat) and the nitrosative agent diethylenetriamine nitric oxide adduct (DETA/NO) were investigated. As one of the cellular consequences of nitrosative stress is alkylation and thereby damage of the DNA, we also investigated the response of Mtb to the alkylating agent methylnitronitrosoguanidine (MNNG). Furthermore, we studied the transcriptomic response of Mtb to mitomycin C (MMC), a substance known to induce DNA cross-linking leading to DSBs [19]. In addition to identifying the DE genes encoding mRNAs, this study aims to detect ncRNA transcripts which are expressed under different stress conditions and to predict their potential target genes. This analysis will enable improved understanding of Mtb regulatory networks that operate under various forms of genotoxic stress.

## Results

### Nitrosative stress induced the most prominent transcriptomic response

**Fig. 1:**
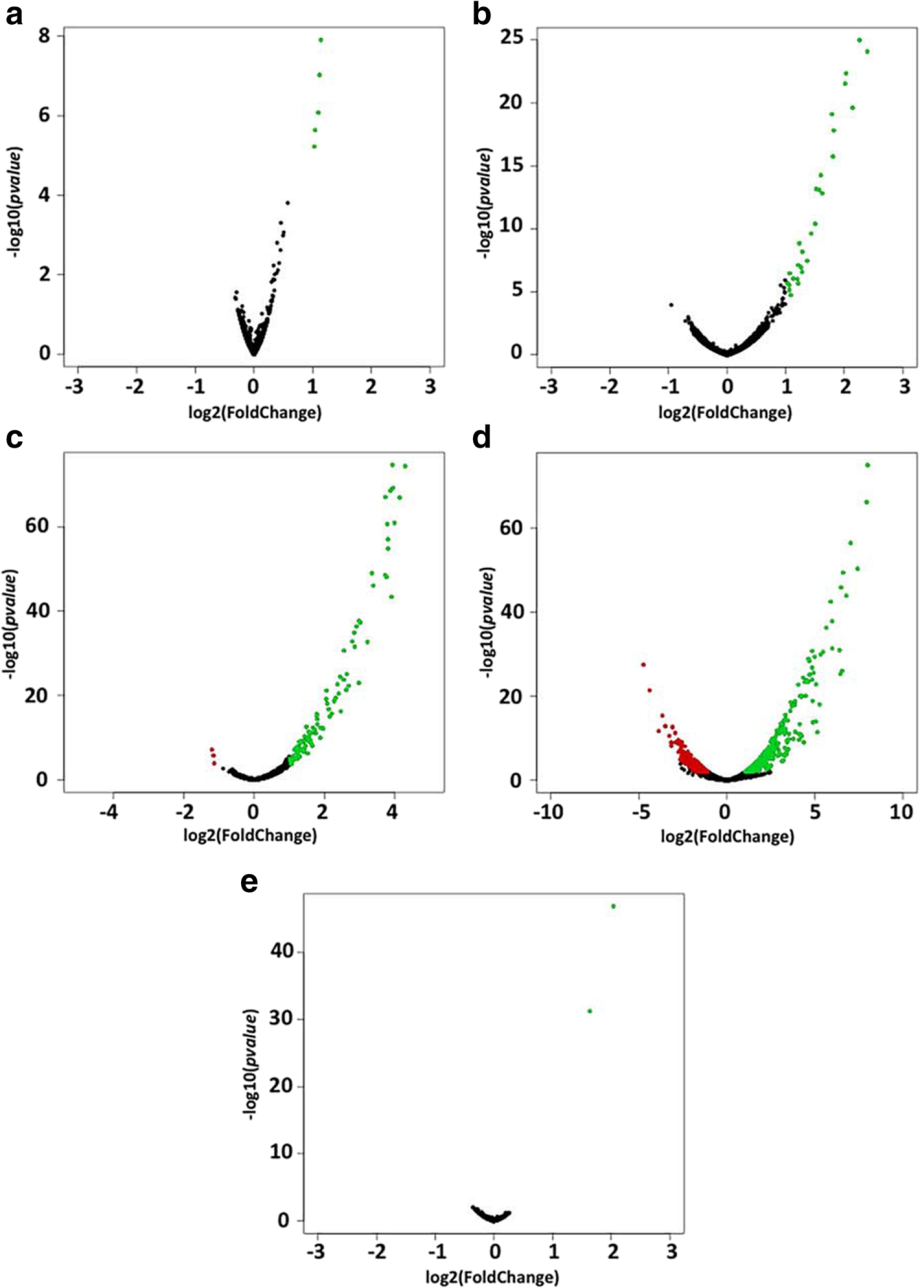
Volcano plots of differentially expressed (DE) genes under five stress conditions. On the x-axis the changes in expression for all genes under stress condition compared to the unstressed condition for **a** paraquat, **b** H_2_O_2,_ **c** MMC, **d** DETA/NO, and **e** MNNG with the *p*-values on the y-axis. Genes with a fold change greater than 2 and a *p*-value ≤ 0.05 were considered as DE. Genes that do not fulfil these criteria are shown in *black*. *Green dots* correspond to up-regulated genes while *red dots* correspond to down-regulated genes under stress

Several DE genes were identified for each genotoxic stress condition investigated. Treatment of Mtb with paraquat, H_2_O_2_ and MNNG caused only the up-regulation of genes. Oxidative stress generated by paraquat led to the up-regulation of five genes namely, *katG, kmtR*, *Rv3269*, *Rv1405c*, and *mmsA* (Fig. 1a; Additional file 1). The genotoxic stresses exerted by H_2_O_2_, MMC and DETA/NO on Mtb led to more pronounced transcriptomic perturbations with 52, 131 and 723 DE genes, respectively (Fig. 1b-d; Additional file 1), while only two DE genes, *alkA* and *ogt* were detected under MNNG treatment (Fig. 1e; Additional file 1).

Up-regulation of 128 genes and the down-regulation of the *rrs* gene, encoding 16S rRNA, and the two ncRNAs MTS2823 and *ssr* were observed under MMC treatment (Additional file 1). Nitrosative stress in the form of DETA/NO resulted in the up-regulation of 383 genes and the down-regulation of 340 genes with fold changes ranging from +257 to -27 (Additional file 1).

In order to validate the RNA-seq data analysis, a small subset of the DE genes identified from each genotoxic stress was selected based on the level of their expression fold change compared to the control. The expression levels were tested using quantitative real-time PCR (qPCR) (Additional file 2). The qPCR results confirmed the results from the deep sequencing approach (Additional file 3: Figure S1).

### Involvement of 3R genes, the type VII secretion system, and *PE/PPE* genes in the genotoxic response

**Fig. 2:**
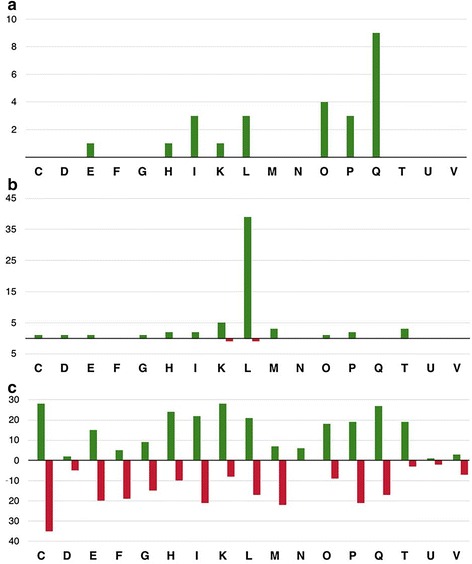
Classification of the differentially expressed (DE) genes according to COG categories. Histograms indicate the number of genes in each COG category. Green colour corresponds to up-regulated genes while red colour corresponds to down-regulated genes. This classification is shown only for (**a**) H_2_O_2_, (**b**) MMC and (**c**) DETA/NO treatment as Paraquat treatment gave only five DE genes (Additional file 1). COG categories: [C] Energy production and conversion, [D] Cell cycle control, cell division, chromosome partitioning, [E] Amino acid transport and metabolism, [F] Nucleotide transport and metabolism, [G] Carbohydrate transport and metabolism, [H] Coenzyme transport and metabolism, [I] Lipid transport and metabolism, [K] Transcription, [L] Replication, recombination and repair, [M] Cell wall/membrane/envelope biogenesis, [N] Cell motility, [O] Post-translational modification, protein turnover, and chaperones, [Q] Secondary metabolites biosynthesis, transport, and catabolism, [T] Signal transduction mechanisms, [U] Intracellular trafficking, secretion, and vesicular transport, [V] Defense mechanisms

A Clusters of orthologous group (COG) analysis of the DE genes from each genotoxic stress was carried out to identify any bias in gene-functional-category distribution. Oxidative stress inflicted by H_2_O_2_ induced the expression of genes belonging to 8 COG categories (Fig. 2a). The top three of these categories includes replication, recombination and repair (3R) genes (COG L), post-translational modifications, protein turnover and chaperones (COG O) and secondary metabolites biosynthesis, transport and catabolism (COG Q) (Fig. 2a). Among the 128 up-regulated genes due to the MMC treatment, 38 come under the COG L category (Fig. 2b). For the DETA/NO stress, the DE genes were distributed in 17 COG categories whereby nine of them contained more down-regulated genes than up-regulated genes (Fig. 2c). The energy production and conversion category (COG C) included the most genes affected with 28 up- and 35- down-regulated genes (Fig. 2c; Additional file 1). The two categories transcription (COG K) and signal transduction mechanisms (COG T) encompassed mostly up-regulated genes. The genes in the categories of nucleotide transport and metabolism (COG F) and cell wall/membrane/envelope biogenesis (COG M) were mostly down-regulated. In COG K, genes encoding sigma factors and SOS response genes were found to be DE. The cell motility category (COG N) contained only 5 up-regulated genes. Four out of these five genes belong to the PE/PPE family *(ppe1, ppe4, ppe20, and ppe37*). Furthermore, the DE genes include members of the T7SS, namely *eccA3, eccC3, eccD1, eccD3* and *eccE3*. Interestingly, among the up-regulated genes, one transcription factor (Rv3249c) was already predicted to regulate the expression of multiple genes [20], including *alkB, rubA* and *rubB,* but also *eccB1 eccB2, eccB3,* and *eccB5*. The proteins EccB1, EccB2, EccB3 and EccB5 are all part of the T7SS.

**Fig. 3:**
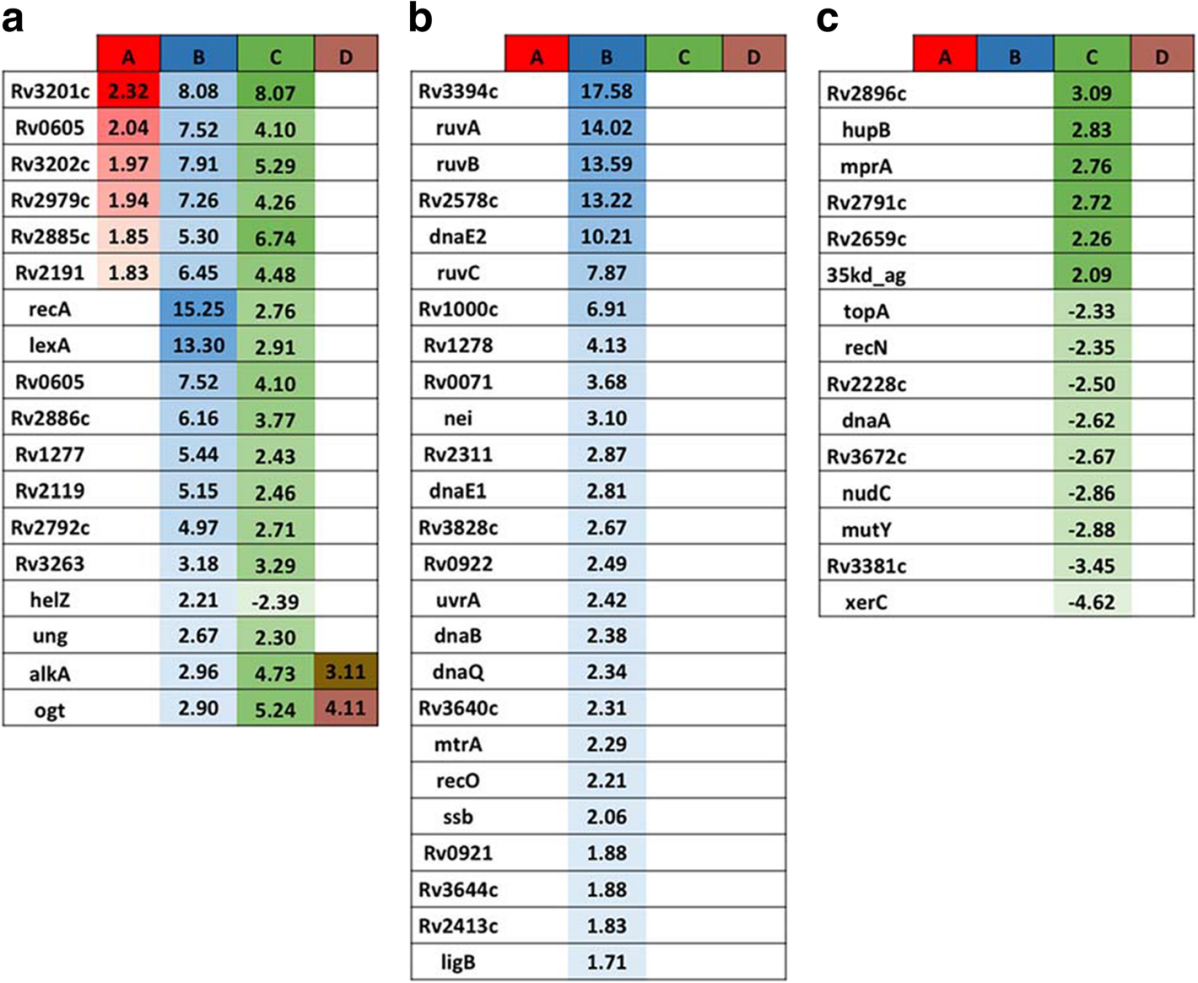
Differentially expressed (DE) 3R genes under various forms of genotoxic stress. Among all DE expressed genes, 58 genes belong to the 3R category. The Table **a** shows the 3R genes that were DE under at least two genotoxic stresses. The Tables **b** and **c** show the DE genes under only one genotoxic stress. H_2_O_2_, **B** MMC, **C** DETA/NO, **D** MNNG

Among the DE 3R genes, predominantly affected by MMC and DETA/NO treatments, are mostly genes that encode helicases, endonucleases and resolvases and have transcription fold changes ranging from -5 to +18 (Fig. 3). The proteins encoded by these 3R genes are directly involved in genome maintenance.

### Identification of new potential 3R genes

For all DE genes annotated as coding for HPs (Additional file 4), we performed 3D structure predictions using Phyre^2^ [21, 22]. By considering only proteins modelled with more than 70 % identity and 30 % coverage, we detected proteins that are predicted to be hydrolases/endonucleases, metal binding proteins, and DNA binding proteins. Some proteins were predicted with a high level of confidence to contain a DNA binding domain (Additional file 4). For example, 3D structural modelling of the protein encoded by *Rv3074*, that was up-regulated 18.3 fold under MMC stress and 2.3 fold under DETA/NO stress, suggests that it probably is a hydrolase/endonuclease.

### Clustering of DE genes into transcription units

**Fig. 4:**
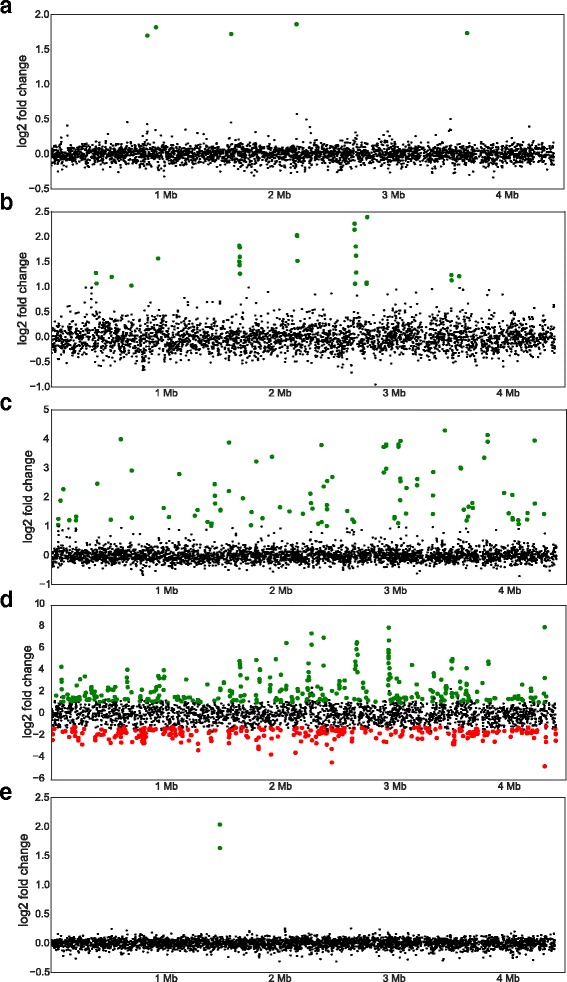
Mapping of the fold change of each gene according to its genomic location. Each dot corresponds to one gene. The x-axis corresponds to the genomic coordinates and the y-axis to the log_2_ fold change. Significantly up-regulated genes are indicated with *green dots* while significantly down-regulated genes are indicated by *red dots*. **a** Paraquat, **b** H_2_O_2_, **c** MMC, **d** DETA/NO, and **e** MNNG

By analysing the distribution of DE genes throughout the genome, we found that several DE genes were organized in clusters, defined as at least two neighbouring genes according to their genomic location (Fig. 4; Additional file 5). Genes in clusters may be part of the same transcriptional unit. No clusters were identified for the DE genes detected in the sample from the paraquat treatment (Fig. 4a). Regarding H_2_O_2_ stress, 5 clusters were identified corresponding to 17 DE genes. The largest cluster (cluster 2) includes 6 genes (*mbtG, mbtF, mbtE, mbtD, mbtC, mbtB*) belonging to the secondary metabolites biosynthesis, transport and catabolism category (COG G). They are involved in the biosynthesis of mycobactin, a mycobacterial siderophore with high affinity for environmental iron [23]. Of the 15 clusters identified for MMC treatment, nine of them contain 3R genes. The cluster 10 is the largest with 4 genes (*Rv2734*, *Rv2735c*, *recX*, and *recA*). Among the 723 DE genes due to the DETA/NO stress, 294 (40.6 %) of them were organized in clusters. The three biggest clusters, cluster 15, cluster 73 and cluster 90 include 19, 13 and 11 DE genes, respectively. The cluster 15 contains ribosomal binding proteins (*rpsJ* - *rplO*), the cluster 73 includes genes encoding for the subunits of the NADH dehydrogenase I (*nuoB* to *nuoN*) and the cluster 90 contains the genes encoding Esx-3 and its secreted substrates. All genes in the cluster 15 and the cluster 73 were down-regulated and the genes in cluster 90 were up-regulated under nitrosative stress. The *mbt* genes were also found to be highly up-regulated under nitrosative stress (Cluster 57 and Cluster 89). As already reported by Yang and colleagues [24], the two up-regulated genes *alkA* and *ogt* due to MNNG stress are located in one cluster (Fig. 4e). Sigma factor binding site analysis in the 100 bp upstream of all 110 identified clusters revealed the presence of binding sites for SigB, SigC, SigE and SigF. The most common was the SigB binding site, found for 21gene clusters, while the binding sites for the other sigma factors were only found once each (Additional file 5).

### Potential role of ncRNA for genotoxic stress response

**Fig. 5:**
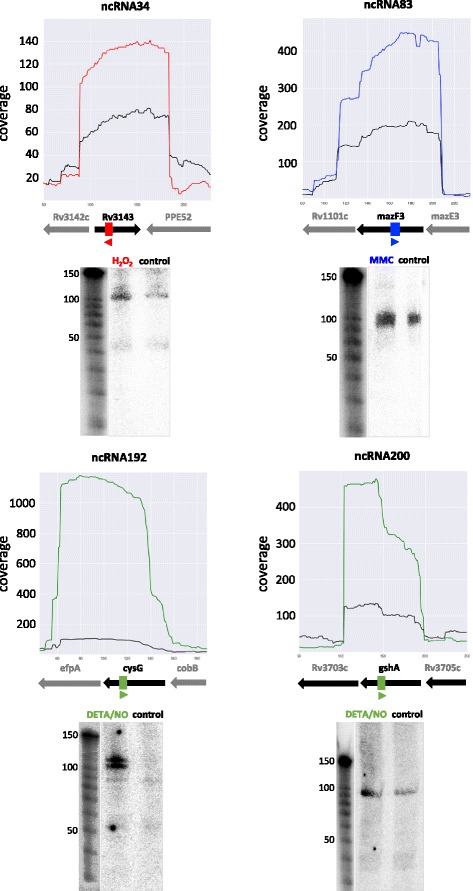
Validation of selected ncRNAs by northern blot. Four newly discovered ncRNAs (ncRNA34, ncRNA83, ncRNA192, ncRNA200) were randomly selected for northern blot validation. For each selected ncRNA, the normalized coverage plots from RNA-seq data of aligned reads is shown on the *top* with the genomic context identified from the Mtb H37Rv (NC_000962.3) genome and the position of the ncRNA is indicated by coloured arrows. The coverage data is indicated with the following colours: no stress -*black*; H_2_O_2_ - *red*; MMC -*blue*; DETA/NO -*green*. RNA samples for the northern blots are from the correspondent samples. Control RNA is from non-stressed cells. For each northern blot, the loading control (5S rRNA) and decade marker are shown

**Fig. 6:**
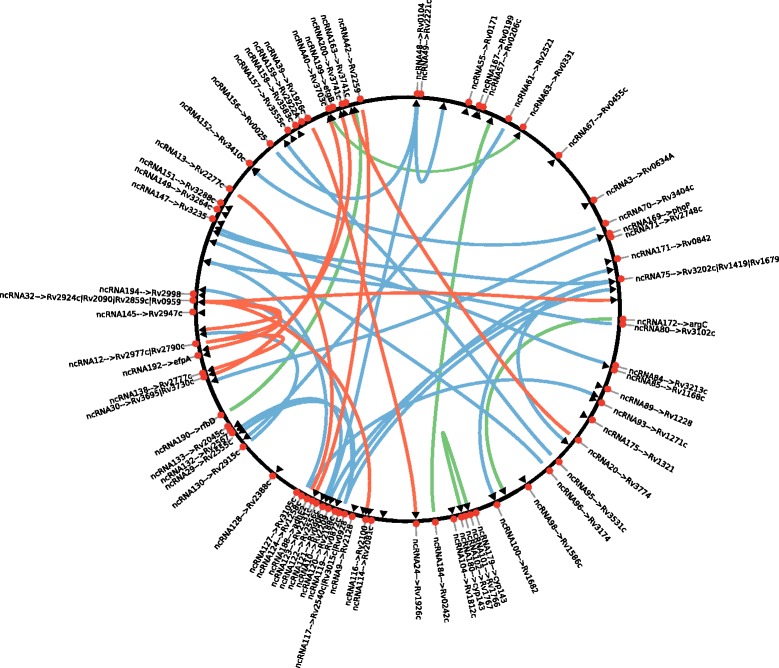
Genomic distribution of identified DE ncRNAs under the different genotoxic stress and their predicted targets. The *circle* represents the Mtb genome. *Red circles* indicate the location of ncRNAs, labelled with their ID. *Triangles* show the location of the targets. Interconnecting lines link between each ncRNA and its corresponding target(s). Different colours reflect the different genotoxic stresses. *Red lines*: oxidative stress. *Green lines*: nitrosative stress. *Blue lines*: MMC stress

Thorough bioinformatics analysis of the sequencing data from the samples treated with H_2_O_2_ and paraquat, 44 and 17 ncRNAs were predicted, respectively (Additional file 6). Out of these, 16 ncRNAs were found under both oxidative stress conditions. Only one ncRNA was identified in the data from the MNNG stressed sample. The data from the DETA/NO stressed samples revealed 36 ncRNAs. From the 120 ncRNAs identified in the data from the MMC stressed sample, we found 93 to be novel (Additional file 6). Among all the 202 ncRNAs identified 44 were previously reported (Additional file 6, highlighted in yellow) [25–29]. The length of the ncRNAs varied between 50 and 270 bases. A subset of the identified ncRNAs was experimentally verified by northern blot analyses confirming the predicted transcript sizes (Fig. 5). By using intaRNA, the potential gene targets of the ncRNAs were predicted. A total of 76 ncRNAs were predicted to regulate 80 genes under paraquat (6), H_2_O_2_ (17), MMC (51) and DETA/NO (13) treatments (Fig. 6; Additional file 6) with 5 genes targeted by more than one ncRNA.

## Discussion

In this study, we determined the transcriptomic responses of the Mtb to various forms of genotoxic stress. Our results show massive and complex gene regulation especially for the nitrosative stress inflicted by DETA/NO. Alkylation damage due to MNNG treatment led to the up-regulation of only two genes, *alkA* and *ogt* that are part of the ada operon of Mtb and this result corroborates a previous study [24].

When facing oxidative stress by H_2_O_2_ or by paraquat, Mtb responded differently in terms of the number of DE genes. However, regarding the ncRNAs, 16 ncRNAs are common under both treatments. Regarding gene regulation, the catalase-peroxidase gene *katG* was found to be the only common gene up-regulated under both treatments. This was also the case under nitrosative stress, confirming the importance of KatG in stress responses [30]. The enzyme KatG, together with the metalloenzyme KmtR, is involved in the oxidative stress response by playing a role in the detoxification of host-generated free radicals [31–33]. In addition, *furA* is up-regulated under oxidative stress due to H_2_O_2_ (fold change = 2.87) and nitrosative (fold change = 4.5) stress conditions. Both oxidizing treatments caused the up-regulation of genes that are mainly taken part in metal transport. The correlation between oxidative stress and expression of metal transport proteins has already been observed [34, 35]. FurA is known to be involved in iron homeostasis and stress response in many bacteria [36, 37]. We suggest that FurA might contribute as an oxidative stress sensing regulator. Our observation of the up-regulation of *mbt* genes under both oxidative and nitrosative stress corroborate the study reported by Voskuil and colleagues [6]. The *mbt* genes participate in the biosynthesis of the hydroxyphenyloxazoline-containing mycobactins, a class of siderophores involved in iron acquisition [38–40]. Iron homeostasis must be finely controlled as both excess and limitation of iron may cause oxidative stress [41–43]. Under normal conditions, the regulation of the siderophore biosynthesis genes is dependent on the iron concentration in the environment which is fluctuating due to a continuous oxidation/reduction of Fe^2+^/Fe^3+^ [44]. The up-regulation of *the mbt* genes demonstrates that Mtb is using this pathway to limit the oxidative stress damage by scavenging more iron from its environment. In addition, the genes *mmpS4, mmpS5, mmpL3-L5*, whose products are needed to transport and export siderophores [40], were up-regulated by more than two fold changes. Concomitantly, the gene *bfrB,* which encodes the iron storage protein bacterioferritin [43], was found to be down-regulated nearly 27 fold under nitrosative stress. These observations indicate that, especially under nitrosative stress, Mtb strongly reacts by acquiring more iron from its environment.

The high number of DE genes under nitrosative stress is probably linked to the up-regulation of several transcription factors from COG K category. Several genes that are part of the T7SS were also up-regulated. A genome-wide binding approach of all predicted transcription factors showed that these genes could be regulated by the transcription factor encoded by Rv3249c [20] that was up-regulated by 7 fold under nitrosative stress. The T7SS, a mycobacterial specialised secretion machinery, translocates different effectors throughout the cell wall [45, 46] and plays a major role in the virulence of Mtb [45, 47]. Of the five T7SSs in Mtb (ESX-1 to ESX-5), ESX-1 [48] and ESX-3 [49, 50] are known to be essential for virulence and growth of Mtb. Components of the T7SS might represent good targets for anti-tuberculosis drug discovery [51]. The newly discovered up-regulation of several ESX-3 core channel genes and the genes encoding for ESX-3 secreted substrates, *pe5*, and *ppe4* (Cluster 90), might be a consequence of the iron depletion under nitrosative stress. To the best of our knowledge this is the first observation that nitrosative stress affects the expression of T7SS genes in Mtb. Interestingly, the secreted substrates PE5 and PPE4 are found to be critical for the siderophore- mediated iron-acquisition functions of ESX-3 while ESXG and EsxH play an essential role in virulence [52]. Eight *pe/ppe* genes were up-regulated with *ppe37* being the most up-regulated one (131 fold). This result corroborates previous studies reporting the over-expression of *ppe37* under nitrosative and oxidative growth conditions [53, 54]. In murine macrophages infected with *M. smegmatis,* PPE37 induced the low production of tumour necrosis factor alpha (TNFα) and interleukin 6 (IL6) [55]. Some of the gene products of the up-regulated *pe*/*ppe* genes might therefore be involved in regulating cytokine production of the host cell. It is also important to highlight that under DETA/NO stress *dosR* and *dosS* in cluster 71 were up-regulated 30 and 18 fold, respectively. The genes *dosR* and *dosS* are conserved in many mycobacterial species and DosS/DosR represents the two-component system that regulates the dormancy regulon in Mtb [56]. The up-regulation of *dosS/dosR* would lead to slower bacterial growth and lower metabolic activity. By reducing its metabolic activity, Mtb could circumvent the genotoxic stress. While the DosS/DosR two component system is extensively studied [56–59] its selective involvement in only some stress reactions needs further investigation.

Notably, DSB damage inflicted by MMC treatment led especially to the up-regulation of 3R genes, predominantly DNA repair genes (Fig. 3 and Additional file 6). Among the up-regulated 3R genes *recA* and *lexA* were the highest up-regulated genes which are in agreement with the fact that bacteria respond to DNA damage by mounting a coordinated cellular SOS response, governed by the RecA and LexA. During normal bacterial growth, the LexA repressor down-regulates its own expression and regulates, in addition, the expression of more than 40 unlinked genes [60]. RecA protein, upon DNA damage, binds to single-stranded DNA and induces the self-cleavage of LexA [61]. The dissociation of LexA from its DNA targets causes the de-represseion of genes for DNA damage repair and tolerance. Among the up-regulated genes, we found nearly 20 genes (Additional file 1, highlighted in blue) that harbour a putative SOS box [62] and thereby could be regulated by LexA. Taken together, this shows that MMC induces a classic SOS response in Mtb.

Several DE genes that encode for HPs were found in our analyses. The 3D structural modelling of these proteins showed that some of them may code for DNA binding proteins, metal binding proteins, or endonucleases. They could play an important role in genome maintenance and stability during genotoxic stress as some of them were strongly induced (more than 20 fold) (Additional file 4). These HPs will be the subjects of future functional characterisation.

While the DETA/NO stress gave the highest number of DE mRNAs, the highest number of stress induced ncRNAs was predicted for MMC stress when compared to the other treatments. Collectively, these results show that Mtb responds very differently to the various forms of stress [4]. Among the cumulative 202 ncRNAs, 19 (9.4 %) 3′ UTRs and 34 (16.8 %) 5′ UTRs were identified. The rest of the ncRNAs (73.8 %) were located in coding sequences. This distribution could be explained by the fact that more than 90 % of the Mtb genome is occupied by coding sequence. The ncRNAs identified are distributed throughout the genome, while there seems to be some ncRNA clustering (red dots in Fig. 6). Local genome content and architecture could be involved [63]. The classification of the predicted target genes (triangles in Fig. 6) showed that six belong to the 3R category indicating an involvement in DNA repair. Among the genes targeted under oxidative stress by ncRNAs are *mutM* (*fpg*), encoding a DNA glycosylase [64], and Rv2090, encoding an exonuclease. Another targeted gene, Rv3202c (*adnA*), was found to be up-regulated under oxidative, nitrosative and MMC stress. This reflects the importance of *adnA*, which was previously reported to be up-regulated together with Rv3201c (*adnB*) under MMC treatment [65]. AdnAB is a helicase-nuclease which is involved in RecA-dependent homologous recombination [11].

## Conclusions

This study demonstrated the distinct transcriptomic responses Mtb exerts when subjected to genotoxic stress. The data analyses revealed that in addition to the already reported DE genes, genes involved in the T7SS and the *pe*/*ppe* gene families were found to be up-regulated under genotoxic stress. The structural analysis of the hypothetical proteins from DE genes suggested that some of them are involved in genome maintenance. We also identified several ncRNAs expressed under stress conditions and their potential gene targets thereby unravelling new regulatory networks of Mtb. The insights into the networks that operate under genotoxic stress will enable deeper understanding of Mtb survival strategies and in turn will help to gain better insight into the mycobacterial response to the host defense. Our results furthermore provide a list of genome maintenance, survival and virulence factors that potentially could be considered as targets in drug discovery and vaccine development.

## Methods

### Cultivation of *Mycobacterium tuberculosis* H37Rv and RNA isolation

Mtb H37Rv was grown in 90 ml Middlebrook 7H9 medium supplemented with albumin-dextrose-catalase (ADC) and 0.05 % Tween 80 at 37 °C with shaking until OD600 was 0.4–0.5. The culture was divided into 9 equal volumes, and the cultures were treated with 10 mM paraquat (Sigma) for 1 h [66], 5 mM H_2_O_2_ (Sigma) for 1 h [6], 0.5 mM DETA/NO (Sigma) for 4 h [6], 3 μM MNNG (Sigma), for 1 h [24], or 20 ng/ml MMC (Sigma) for 24 h [25], respectively. Stock solutions of MNNG and DETA/NO were prepared in DMSO and 0.01 N NaOH, respectively, whereas paraquat and MMC were dissolved in sterile MQ H_2_O. Untreated control cultures were prepared and analyzed in the same manner as the experimental cultures. Cells were harvested by centrifugation and frozen in RNAlater (Life Technologies). Total RNA was isolated using TRIzol (Invitrogen) followed by RNeasy spin columns (Qiagen) as previously described [64] and treated with Turbo DNase (Life Technologies) following the manufacturer’s instruction. Three independent experiments were conducted for each genotoxic stress and the controls. The workflow of methods employed to identify DE genes and non-coding RNAs are depicted in Additional file 7: Figure S2.

### Library preparation for cDNA and sequencing

The cDNA library construction and pyrosequencing were performed as previously described [67]. RNA sequencing was carried out using the HiSeq 2000 sequencing system (Illumina) at the Max Planck Genome Centre, Cologne, Germany. For each sample a read depth ranging from 5 to 10 million reads was obtained.

### Processing of RNAseq data and bioinformatics analysis

The workflow for bioinformatics data processing of RNAseq data is outlined in Additional file 7: Figure S2. Quality control was performed using the fastx-toolkit [68] and FastQC [69]. Reads with mean quality score lower than 30 were excluded from downstream analysis. Reads were aligned against the Mtb H37Rv genome sequence (version NC_000962.3) as a reference, using segemehl [70]. The segemehl implements an enhanced suffix array (ESA) matching strategy, yielding higher sensitivity and lower false positive rate than the Burrows-Wheeler Aligner (BWA) when aligning RNA-seq data [71]. The number of reads corresponding to each annotated gene was determined using the HTSeq python package by applying the ‘union’ overlap resolution mode [72]. Data were normalized and DE genes were identified using DESeq2 package [73] from the Bioconductor framework release 3.0 [74, 75]. For normalization, a scaling factor for each RNA-seq data was calculated as the median of the ratio of read count (for each gene) divided by the geometric mean across all generated RNA-seq data. Raw read counts were divided by the scaling factor. A fold-change in expression equal or higher than 2 with a *p*-value < 0.05 was considered as significant, and genes fulfilling these criteria were considered to be DE genes. For DE genes coding for HPs, structural modelling was done using Phyre^2^ [21, 22].

### Sigma factors binding sites distribution and clustered genes

The presence/absence of binding sites for all known sigma factors, SigA-SigH and SigJ-SigM [76–81] were identified. For each binding site, the corresponding consensus sequence was retrieved and converted in python regular expressions. These regular expressions were used to check the presence/absence of each binding site in a region of 100 bp upstream of each identified genes organized in clusters.

### Identification of non-coding RNAs

Putative ncRNAs were identified with Rockhopper [82, 83] using default parameters for strand-specific reads, and then manually verified after visualization using Rockhopper viewer and COV2HTML [84]. In addition, a python script written using the two libraries matplotlib [85] and seaborn [86] to analyse and plot the coverage rate using different sizes of sliding windows across the reads alignment for the control and treated samples. All scripts for data management and analyses were written in-house in Python or R.

### Prediction of ncRNA targeted regions

The potentially targeted regions of small RNAs (sRNA) were predicted using IntaRNA [87–89]. This method takes into account the secondary structure of the ncRNA, the mRNA target candidate and their hybridization energy. Only candidates with a *p-value* < 0.05 and a false discovery rate (FDR) ≤ 0.1 were considered to be potentially significant. Subsequently, putative target genes of these ncRNAs were classified according to the COG classification system [90].

### Northern blotting of ncRNA

Validation of ncRNA was done by northern blotting. Total RNA (15–20 μg/per lane) was resolved on a 8 % denaturing polyacrylamide gel containing 8 M urea for 75 min at 300 V. The RNA was electroblotted onto nylon membrane (BrightStar plus, Ambion) using the Trans-Blot Turbo transfer system (BioRad). After UV-crosslinking in an ultraviolet crosslinker (CL1000, UVP Inc), the membranes were stained with 0.03 % methylene blue in 0.3 M sodium acetate to visualize the RNA bands and control the transfer. Then the membranes were pre-hybridized in Ultrahyb ultrasensitive hybridization buffer (Ambion) for 1 h at 68 °C. Riboprobes were synthesized using the mirVana miRNA Probe construction kit (Ambion) and labelled with [α-32P]-UTP. Labelled riboprobes complementary to each ncRNA target (Additional file 8) were added and incubated at 68 °C overnight. After washing twice in low stringency buffer (Ambion) for 15 min and once with high stringency buffer (Ambion) for 15 min, the membranes were exposed to phosphorimaging screens and the screens were scanned using a phosphorimager 445 SI (Amersham). Decade marker (10–150 nucleotides, Ambion) and RNA century marker (100–500 nucleotides, Ambion) were used as size standards.

## Additional files

Additional file 1: List of differentially expressed (DE) genes encoding mRNA under each genotoxic stress. (XLSX 96 kb)
Additional file 2: List of oligos used for the validation of DE genes and RT qPCR procedure. (DOCX 16 kb)
Additional file 3: Figure S1. Validation of selected differentially expressed (DE) genes. The graphs represent the fold differences in the level of expression of 7 DE-genes after cell exposure to paraquat and H_2_O_2_ (red), MMC (blue) and DETA/NO (green), relative to the non-treated sample. The error bars indicate the standard deviation. The *p*-values were calculated with a paired Student’s t-test with two-tailed distribution and are indicated; * *p*-value ≤ 0.05; ** *p*-value ≤ 0.01. Horizontal black bars reflect the fold change values from RNA-seq data. (EPS 441 kb)
Additional file 4: Functional prediction of DE hypothetical proteins. (XLSX 36 kb)
Additional file 5: Clustering of DE genes encoding mRNA. (XLSX 40 kb)
Additional file 6: Identified ncRNAs under each genotoxic stress. (XLSX 32 kb)
Additional file 7: Figure S2. Workflow showing the methods employed to identify differentially expressed genes encoding mRNA and ncRNAs. Schematic representation of the followed steps from reads analyses to differentially expressed mRNA and ncRNAs discovery. (EPS 588 kb)
Additional file 8: List of oligos used for riboprobe construction. (DOCX 14 kb)

## Abbreviations

COG: clusters of orthologous group
DE: Differentially expressed
DETA/NO: Diethylenetriamine/nitric oxide adduct
DSBs: Double strand breaks
HPs: Hypothetical proteins
MMC: Mitomycin C
MNNG: N-methyl-N-nitro-N-nitrosoguanidine
Mtb: *Mycobacterium tuberculosis*
ncRNA: Non-coding RNA
PE/PPE: Proline-glutamic acid and proline-proline-glutamic acid
RNS: Reactive nitrogen species
ROS: Reactive oxygen species
T7SS: Type VII secretion system

## References

[1] http://www.who.int/tb/publications/global_report/en/: 2015.

[2] Flynn JL, Chan J (2001). Tuberculosis: latency and reactivation. Infect Immun.

[3] Co DO, Hogan LH, Kim SI, Sandor M (2004). Mycobacterial granulomas: keys to a long-lasting host-pathogen relationship. Clin Immunol.

[4] Ehrt S, Rhee K, Schnappinger D (2015). Mycobacterial genes essential for the pathogen's survival in the host. Immunol Rev.

[5] Dosanjh NS, Rawat M, Chung JH, Av-Gay Y (2005). Thiol specific oxidative stress response in Mycobacteria. FEMS Microbiol Lett.

[6] Voskuil MI, Bartek IL, Visconti K, Schoolnik GK (2011). The response of mycobacterium tuberculosis to reactive oxygen and nitrogen species. Front Microbiol.

[7] Lamichhane G (2011). Mycobacterium tuberculosis response to stress from reactive oxygen and nitrogen species. Front Microbiol.

[8] Firmani MA, Riley LW (2002). Reactive nitrogen intermediates have a bacteriostatic effect on Mycobacterium tuberculosis in vitro. J Clin Microbiol.

[9] Adams LB, Dinauer MC, Morgenstern DE, Krahenbuhl JL (1997). Comparison of the roles of reactive oxygen and nitrogen intermediates in the host response to Mycobacterium tuberculosis using transgenic mice. Tubercle and Lung Disease: The Official Journal of the International Union Against Tuberculosis and Lung Disease.

[10] Nathan C, Shiloh MU (2000). Reactive oxygen and nitrogen intermediates in the relationship between mammalian hosts and microbial pathogens. Proc Natl Acad Sci U S A.

[11] Gupta R, Barkan D, Redelman-Sidi G, Shuman S, Glickman MS (2011). Mycobacteria exploit three genetically distinct DNA double-strand break repair pathways. Mol Microbiol.

[12] Han Y, Liu L, Fang N, Yang R, Zhou D (2013). Regulation of pathogenicity by noncoding RNAs in bacteria. Future Microbiol.

[13] Storz G, Vogel J, Wassarman KM (2011). Regulation by small RNAs in bacteria: expanding frontiers. Mol Cell.

[14] Arnvig K, Young D (2012). Non-coding RNA and its potential role in Mycobacterium tuberculosis pathogenesis. RNA Biol.

[15] Waters LS, Storz G (2009). Regulatory RNAs in bacteria. Cell.

[16] Papenfort K, Vogel J (2010). Regulatory RNA in bacterial pathogens. Cell Host Microbe.

[17] Repoila F, Darfeuille F (2009). Small regulatory non-coding RNAs in bacteria: physiology and mechanistic aspects. Biol Cell.

[18] Park H-D, Guinn KM, Harrell MI, Liao R, Voskuil MI, Tompa M, Schoolnik GK, Sherman DR (2003). Rv3133c/dosR is a transcription factor that mediates the hypoxic response of Mycobacterium tuberculosis. Mol Microbiol.

[19] Noll DM, Mason TM, Miller PS (2006). Formation and repair of interstrand cross-links in DNA. Chem Rev.

[20] Minch KJ, Rustad TR, Peterson EJ, Winkler J, Reiss DJ, Ma S, Hickey M, Brabant W, Morrison B, Turkarslan S (2015). The DNA-binding network of Mycobacterium tuberculosis. Nat Commun.

[21] Kelley LA, Mezulis S, Yates CM, Wass MN, Sternberg MJ (2015). The Phyre2 web portal for protein modeling, prediction and analysis. Nat Protoc.

[22] Kelley LA, Sternberg MJ (2009). Protein structure prediction on the Web: a case study using the Phyre server. Nat Protoc.

[23] De Voss JJ, Rutter K, Schroeder BG, Barry CE (1999). Iron acquisition and metabolism by mycobacteria. J Bacteriol.

[24] Yang M, Aamodt RM, Dalhus B, Balasingham S, Helle I, Andersen P, Tonjum T, Alseth I, Rognes T, Bjoras M (2011). The ada operon of Mycobacterium tuberculosis encodes two DNA methyltransferases for inducible repair of DNA alkylation damage. DNA Repair (Amst).

[25] Arnvig KB, Young DB (2009). Identification of small RNAs in Mycobacterium tuberculosis. Mol Microbiol.

[26] Arnvig KB, Cortes T, Young DB (2014). Noncoding RNA in Mycobacteria. Microbiol Spectr.

[27] Miotto P, Forti F, Ambrosi A, Pellin D, Veiga DF, Balazsi G, Gennaro ML, Di Serio C, Ghisotti D, Cirillo DM (2012). Genome-wide discovery of small RNAs in Mycobacterium tuberculosis. PLoS One.

[28] Haning K, Cho SH, Contreras LM (2014). Small RNAs in mycobacteria: an unfolding story. Front Cell Infect Microbiol.

[29] Wang M, Fleming J, Li Z, Li C, Zhang H, Xue Y, Chen M, Zhang Z, Zhang XE, Bi L (2016). An automated approach for global identification of sRNA-encoding regions in RNA-Seq data from Mycobacterium tuberculosis. Acta Biochim Biophys Sin Shanghai.

[30] Mulder MA, Nair S, Abratt VR, Zappe H, Steyn LM (1999). Involvement of the N- and C-terminal domains of Mycobacterium tuberculosis KatG in the protection of mutant Escherichia coli against DNA-damaging agents. Microbiology.

[31] Milano A, Forti F, Sala C, Riccardi G, Ghisotti D (2001). Transcriptional regulation of furA and katG upon oxidative stress in Mycobacterium smegmatis. J Bacteriol.

[32] Pagan-Ramos E, Song J, McFalone M, Mudd MH, Deretic V (1998). Oxidative stress response and characterization of the oxyR-ahpC and furA-katG loci in Mycobacterium marinum. J Bacteriol.

[33] Gupta S, Chatterji D (2005). Stress responses in mycobacteria. IUBMB Life.

[34] Agranoff D, Krishna S (2004). Metal ion transport and regulation in Mycobacterium tuberculosis. Frontiers in Bioscience: A Journal and Virtual Library.

[35] Neyrolles O, Wolschendorf F, Mitra A, Niederweis M (2015). Mycobacteria, metals, and the macrophage. Immunol Rev.

[36] Sala C, Forti F, Di Florio E, Canneva F, Milano A, Riccardi G, Ghisotti D (2003). Mycobacterium tuberculosis FurA autoregulates its own expression. J Bacteriol.

[37] Eckelt E, Meissner T, Meens J, Laarmann K, Nerlich A, Jarek M, Weiss S, Gerlach GF, Goethe R (2015). FurA contributes to the oxidative stress response regulation of Mycobacterium avium ssp. paratuberculosis. Front Microbiol.

[38] De Voss JJ, Rutter K, Schroeder BG, Su H, Zhu Y, Barry CE (2000). The salicylate-derived mycobactin siderophores of Mycobacterium tuberculosis are essential for growth in macrophages. Proc Natl Acad Sci U S A.

[39] Quadri LE, Sello J, Keating TA, Weinreb PH, Walsh CT (1998). Identification of a Mycobacterium tuberculosis gene cluster encoding the biosynthetic enzymes for assembly of the virulence-conferring siderophore mycobactin. Chem Biol.

[40] Wells RM, Jones CM, Xi Z, Speer A, Danilchanka O, Doornbos KS, Sun P, Wu F, Tian C, Niederweis M (2013). Discovery of a siderophore export system essential for virulence of Mycobacterium tuberculosis. PLoS Pathog.

[41] Poole K (2012). Bacterial stress responses as determinants of antimicrobial resistance. J Antimicrob Chemother.

[42] Andrews SC, Robinson AK, Rodriguez-Quinones F (2003). Bacterial iron homeostasis. FEMS Microbiol Rev.

[43] Rodriguez GM, Voskuil MI, Gold B, Schoolnik GK, Smith I (2002). ideR, An essential gene in mycobacterium tuberculosis: role of IdeR in iron-dependent gene expression, iron metabolism, and oxidative stress response. Infect Immun.

[44] Galaris D, Pantopoulos K (2008). Oxidative stress and iron homeostasis: mechanistic and health aspects. Crit Rev Clin Lab Sci.

[45] Houben EN, Korotkov KV, Bitter W (2014). Take five - Type VII secretion systems of Mycobacteria. Biochim Biophys Acta.

[46] Simeone R, Bottai D, Frigui W, Majlessi L, Brosch R (2015). ESX/type VII secretion systems of mycobacteria: Insights into evolution, pathogenicity and protection. Tuberculosis.

[47] Mendum TA, Wu H, Kierzek AM, Stewart GR (2015). Lipid metabolism and Type VII secretion systems dominate the genome scale virulence profile of Mycobacterium tuberculosis in human dendritic cells. BMC Genomics.

[48] Abdallah AM, van Gey Pittius NC, Champion PA, Cox J, Luirink J, Vandenbroucke-Grauls CM, Appelmelk BJ, Bitter W (2007). Type VII secretion--mycobacteria show the way. Nat Rev Microbiol.

[49] Serafini A, Boldrin F, Palu G, Manganelli R (2009). Characterization of a Mycobacterium tuberculosis ESX-3 conditional mutant: essentiality and rescue by iron and zinc. J Bacteriol.

[50] Sassetti CM, Boyd DH, Rubin EJ (2003). Genes required for mycobacterial growth defined by high density mutagenesis. Mol Microbiol.

[51] Feltcher ME, Sullivan JT, Braunstein M (2010). Protein export systems of Mycobacterium tuberculosis: novel targets for drug development?. Future Microbiol.

[52] Tufariello JM, Chapman JR, Kerantzas CA, Wong KW, Vilcheze C, Jones CM, Cole LE, Tinaztepe E, Thompson V, Fenyo D (2016). Separable roles for Mycobacterium tuberculosis ESX-3 effectors in iron acquisition and virulence. Proc Natl Acad Sci U S A.

[53] Schnappinger D, Ehrt S, Voskuil MI, Liu Y, Mangan JA, Monahan IM, Dolganov G, Efron B, Butcher PD, Nathan C (2003). Transcriptional Adaptation of Mycobacterium tuberculosis within Macrophages: Insights into the Phagosomal Environment. J Exp Med.

[54] Voskuil MI, Schnappinger D, Rutherford R, Liu Y, Schoolnik GK (2004). Regulation of the Mycobacterium tuberculosis PE/PPE genes. Tuberculosis.

[55] Daim S, Kawamura I, Tsuchiya K, Hara H, Kurenuma T, Shen Y, Dewamitta SR, Sakai S, Nomura T, Qu H (2011). Expression of the Mycobacterium tuberculosis PPE37 protein in Mycobacterium smegmatis induces low tumour necrosis factor alpha and interleukin 6 production in murine macrophages. J Med Microbiol.

[56] Voskuil MI, Schnappinger D, Visconti KC, Harrell MI, Dolganov GM, Sherman DR, Schoolnik GK (2003). Inhibition of respiration by nitric oxide induces a Mycobacterium tuberculosis dormancy program. J Exp Med.

[57] Saini DK, Malhotra V, Dey D, Pant N, Das TK, Tyagi JS (2004). DevR-DevS is a bona fide two-component system of Mycobacterium tuberculosis that is hypoxia-responsive in the absence of the DNA-binding domain of DevR. Microbiology.

[58] Voskuil MI, Schlesinger LS (2015). Toward Resolving the Paradox of the Critical Role of the DosR Regulon in Mycobacterium tuberculosis Persistence and Active Disease. Am J Respir Crit Care Med.

[59] Boon C, Dick T (2012). How Mycobacterium tuberculosis goes to sleep: the dormancy survival regulator DosR a decade later. Future Microbiol.

[60] Courcelle J, Khodursky A, Peter B, Brown PO, Hanawalt PC (2001). Comparative gene expression profiles following UV exposure in wild-type and SOS-deficient Escherichia coli. Genetics.

[61] Little JW (1991). Mechanism of specific LexA cleavage: autodigestion and the role of RecA coprotease. Biochimie.

[62] Davis EO, Dullaghan EM, Rand L (2002). Definition of the mycobacterial SOS box and use to identify LexA-regulated genes in Mycobacterium tuberculosis. J Bacteriol.

[63] Wu CY, Li QZ, Feng ZX (2016). Non-coding RNA identification based on topology secondary structure and reading frame in organelle genome level. Genomics.

[64] Olsen I, Balasingham SV, Davidsen T, Debebe E, Rodland EA, van Soolingen D, Kremer K, Alseth I, Tonjum T (2009). Characterization of the major formamidopyrimidine-DNA glycosylase homolog in Mycobacterium tuberculosis and its linkage to variable tandem repeats. FEMS Immunol Med Microbiol.

[65] Rand L, Hinds J, Springer B, Sander P, Buxton RS, Davis EO (2003). The majority of inducible DNA repair genes in Mycobacterium tuberculosis are induced independently of RecA. Mol Microbiol.

[66] Hu Y, Coates AR (2009). Acute and persistent Mycobacterium tuberculosis infections depend on the thiol peroxidase TpX. PLoS One.

[67] Sittka A, Sharma CM, Rolle K, Vogel J (2009). Deep sequencing of Salmonella RNA associated with heterologous Hfq proteins in vivo reveals small RNAs as a major target class and identifies RNA processing phenotypes. RNA Biol.

[68] http://hannonlab.cshl.edu/fastx_toolkit. In*.*; 2010.

[69] http://www.bioinformatics.babraham.ac.uk/projects/fastqc/. In*.*; 2015.

[70] Hoffmann S, Otto C, Kurtz S, Sharma CM, Khaitovich P, Vogel J, Stadler PF, Hackermuller J (2009). Fast mapping of short sequences with mismatches, insertions and deletions using index structures. PLoS Comput Biol.

[71] Otto C, Stadler PF, Hoffmann S (2014). Lacking alignments? The next-generation sequencing mapper segemehl revisited. Bioinformatics.

[72] Anders S, Pyl PT, Huber W (2015). HTSeq--a Python framework to work with high-throughput sequencing data. Bioinformatics.

[73] Love MI, Huber W, Anders S (2014). Moderated estimation of fold change and dispersion for RNA-seq data with DESeq2. Genome Biol.

[74] Huber W, Carey VJ, Gentleman R, Anders S, Carlson M, Carvalho BS, Bravo HC, Davis S, Gatto L, Girke T (2015). Orchestrating high-throughput genomic analysis with Bioconductor. Nat Methods.

[75] Gentleman RC, Carey VJ, Bates DM, Bolstad B, Dettling M, Dudoit S, Ellis B, Gautier L, Ge Y, Gentry J (2004). Bioconductor: open software development for computational biology and bioinformatics. Genome Biol.

[76] Agarwal N, Tyagi AK (2006). Mycobacterial transcriptional signals: requirements for recognition by RNA polymerase and optimal transcriptional activity. Nucleic Acids Res.

[77] Lee JH, Karakousis PC, Bishai WR (2008). Roles of SigB and SigF in the Mycobacterium tuberculosis sigma factor network. J Bacteriol.

[78] Raman S, Hazra R, Dascher CC, Husson RN (2004). Transcription regulation by the Mycobacterium tuberculosis alternative sigma factor SigD and its role in virulence. J Bacteriol.

[79] Calamita H, Ko C, Tyagi S, Yoshimatsu T, Morrison NE, Bishai WR (2005). The Mycobacterium tuberculosis SigD sigma factor controls the expression of ribosome-associated gene products in stationary phase and is required for full virulence. Cell Microbiol.

[80] Rodrigue S, Brodeur J, Jacques PE, Gervais AL, Brzezinski R, Gaudreau L (2007). Identification of mycobacterial sigma factor binding sites by chromatin immunoprecipitation assays. J Bacteriol.

[81] Raman S, Puyang X, Cheng TY, Young DC, Moody DB, Husson RN (2006). Mycobacterium tuberculosis SigM positively regulates Esx secreted protein and nonribosomal peptide synthetase genes and down regulates virulence-associated surface lipid synthesis. J Bacteriol.

[82] Tjaden B (2015). De novo assembly of bacterial transcriptomes from RNA-seq data. Genome Biol.

[83] McClure R, Balasubramanian D, Sun Y, Bobrovskyy M, Sumby P, Genco CA, Vanderpool CK, Tjaden B (2013). Computational analysis of bacterial RNA-Seq data. Nucleic Acids Res.

[84] Monot M, Orgeur M, Camiade E, Brehier C, Dupuy B (2014). COV2HTML: a visualization and analysis tool of bacterial next generation sequencing (NGS) data for postgenomics life scientists. Omics: A Journal of Integrative Biology.

[85] Hunter JD (2007). Matplotlib: A 2D graphics environment. Computing In Science & Engineering.

[86] https://stanford.edu/~mwaskom/software/seaborn. In*.*; 2015.

[87] Wright PR, Georg J, Mann M, Sorescu DA, Richter AS, Lott S, Kleinkauf R, Hess WR, Backofen R (2014). CopraRNA and IntaRNA: predicting small RNA targets, networks and interaction domains. Nucleic Acids Res.

[88] Smith C, Heyne S, Richter AS, Will S, Backofen R (2010). Freiburg RNA Tools: a web server integrating INTARNA, EXPARNA and LOCARNA. Nucleic Acids Res.

[89] Busch A, Richter AS, Backofen R (2008). IntaRNA: efficient prediction of bacterial sRNA targets incorporating target site accessibility and seed regions. Bioinformatics.

[90] Galperin MY, Makarova KS, Wolf YI, Koonin EV (2015). Expanded microbial genome coverage and improved protein family annotation in the COG database. Nucleic Acids Res.

