# Supplementary material for: The Mycobacterium tuberculosis transcriptional landscape under genotoxic stress
Source: BMC Genomics. 2016 Oct 10;17:791. doi: 10.1186/s12864-016-3132-1 (PMC5057432; doi:10.1186/s12864-016-3132-1)
Supplement: Additional file 2: — List of oligos used for the validation of DE genes and RT qPCR procedure. (DOCX 16 kb) [file 12864_2016_3132_MOESM2_ESM.docx]

**List of oligos used for the validation of differentially expressed genes.**

| Gene | Genotoxic stress | Length of amplicon | Oligo | Sequence 5′🡪3′ |
| --- | --- | --- | --- | --- |
| Rv3269 | Paraquat | 107 | SF267  SF268 | GAATCGGCCCGCCTAAA CAGTGGTCGTGGTCGTG |
| Rv2466c | H_2_O_2_ | 100 | SF269  SF270 | TCAATGGTGTGGCGTTCT GGGTAGGAAGCGAAGGTAAC |
| Rv1907c | H_2_O_2_ | 101 | SF273  SF274 | CTTGCTGACTCCCGGTATG TGGCGATCGCACAATACA |
| Rv3394c | MMC | 90 | SF228SF229 | CGCGCTCAGGTATTGCT  TTCGTAGCTTCCCTCCAGATA |
| Rv3395c | MMC | 79 | SF230SF231 | CGCGGTGATACCAGATCC  GAGCACCACCAGATCCAT |
| Rv2624c | DETA/NO | 77 | SF212SF213 | AGTGATCAAGCCGACACATC  CTTCCCGAAGCGATCTTTCA |
| Rv2625c | DETA/NO | 96 | SF214SF215 | TCTTGATCGCGTTGGGATTG GAGCGGCAGCGAAGATAAA |

## Reverse transcription-quantitative real-time polymerase chain reaction (RT-qPCR)

To confirm putative DE genes RT-qPCR was performed. Total RNA (700 ng) was used as template for cDNA synthesis using high capacity RNA-to-cDNA kit (Applied Biosystems) according to the manufacturer’s instructions. Primers used in RT-qPCR are listed in Additional file 5. The RT-qPCR was carried out using Power SYBR® Green PCR Master Mix kit (Applied Biosystems) in a StepOnePlus Real-Time PCR system (Applied Biosystems). The relative standard curve method was used with cDNA from an untreated sample as the calibrator, and the Mtb *sigA* gene as an internal control. From the two biological replicates the cDNA samples were analysed by qPCR three times using duplicate reactions. The expression of the different genes was normalized to *sigA*.
