# Supplementary material for: The Mycobacterium tuberculosis transcriptional landscape under genotoxic stress
Source: BMC Genomics. 2016 Oct 10;17:791. doi: 10.1186/s12864-016-3132-1 (PMC5057432; doi:10.1186/s12864-016-3132-1)
Supplement: Additional file 8: — List of oligos used for riboprobe construction. (DOCX 14 kb) [file 12864_2016_3132_MOESM8_ESM.docx]

**Additional file 6. List of oligos used for riboprobe* construction.**

| ncRNA | Genotoxic stress | Expected size | Oligo | Sequence 5′🡪3′ |  |  |  |
| --- | --- | --- | --- | --- | --- | --- | --- |
| gshA | DETA/NO | 92 | SVB166 | acgtccgcgcccgcgcccgcgccagatgacaagccagccctgtctc | | | |
| cysG | DETA/NO | 106 | SVB167 | cgccgctcaggccgactacagccccgatcacgatgatcgccctgtctc | | | |
| Rv3143 | H_2_O_2_ | 96 | SVB161 | caccgtcgaggatggccaagtcgatgcccccccgatccctgtctc | | | |
| mazF3 | MMC | 96 | SVB169 | tagagccggctcctgcgacgcgagcaggtagccgattcctgtctc | | | |

*The 3´ends of template oligos are complementary to the T7 promoter oligo supplied in probe construction kit
